# Atomic-Layer-Grown Pt on Textile Boosts Adsorption and Sensitivity of MXene Gel Inks for Wearable Electronics

**DOI:** 10.3390/gels12010019

**Published:** 2025-12-24

**Authors:** Jiahui Li, Yang Zhang, Weidong Song, Zhangping Jin, Tao Lan, Qiuwei Shi, Yannan Xie

**Affiliations:** 1 State Key Laboratory of Flexible Electronics and Information Displays, Institute of Advanced Materials (IAM), College of Materials Science and Engineering, Nanjing University of Posts & Telecommunications, Nanjing 210023, China; 18762267734@163.com (Y.Z.); 1223066533@njupt.edu.cn (W.S.); 1223066444@njupt.edu.cn (Z.J.); 2022060718@njupt.edu.cn (T.L.); iamynxie@njupt.edu.cn (Y.X.); 2 College of Chemistry and Materials Science, Nanjing University of Information Science & Technology, Nanjing 210023, China

**Keywords:** MXene gel ink, atomic layer deposition, platinum growth, surface adsorption, wearable electronics

## Abstract

The reliable integration of high-performance noble metal interfaces with flexible substrates is a key requirement for wearable electronics. However, achieving uniform, mechanically robust and functionally active coatings on fabric surfaces remains highly challenging. This study reports the atomic-layered-deposition (ALD) growth of platinum (Pt) on textile at low temperatures. Through ozone plasma-assisted activation technology, Pt nucleation can be achieved at 100 °C, forming a dense and defect-suppressed Pt layer that substantially increases the surface oxygen functional groups and enhances binding affinity. The resulting Pt layer also significantly enhances the adsorption behavior and sensing performance of Ti_3_C_2_T_x_ MXene gel inks on textile. At the atomic scale, the engineered Pt–MXene interface promotes stronger adsorption of MXene sheets and establishes efficient electron/ion transport pathways within the gel network. Ultimately, the conductive textile treated with Pt functionalized layers (MXene/Pt@textile) exhibits significantly enhanced sensing sensitivity and signal stability, enabling precise detection of human motions, pressure, and subtle physiological vibrations. The synergistic effect of ALD Pt layers and MXene gel inks creates a textile platform combining robustness, breathability, and high responsiveness.

## 1. Introduction

The rapid development of wearable electronics requires textile-based platforms that are soft, breathable, and capable of stable electrical performance during continuous human motion [1,2,3]. However, the intrinsic roughness, chemical inertness, and porous architecture of textile fibers make it difficult to achieve strong adhesion and reliable conductivity when coated with functional materials [4,5,6]. As a result, signal instability and poor mechanical durability remain major challenges for wearable sensing systems [7,8,9].

Two-dimensional (2D) MXene materials, particularly Ti_3_C_2_T_x_, have shown great promise for wearable electronics due to their high electrical conductivity, hydrophilicity, and ease of processing into gel inks [10,11,12,13]. MXene gel inks can conformally coat fabric fibers to form multifunctional sensing layers. Nevertheless, their practical performance is often limited by weak interfacial coupling and insufficient charge transport between MXene flakes and the underlying textile [14,15,16,17]. Mechanical deformation disrupts these weak contacts, leading to sensitivity degradation and unstable long-term operation [18,19,20,21].

To address interfacial limitations, conductive fillers such as silver nanowires, metal nanoparticles, and conductive polymers have been used to reinforce textile substrates [22,23,24,25]. Although these materials improve surface conductivity, they still suffer from significant resistance oscillations during dynamic strain. This instability arises from a fundamental limitation: textile fibers are intrinsically insulating, meaning conductive networks formed only on the surface are easily disrupted by bending or twisting [26,27]. Traditional methods such as surface coating, chemical plating, and physical vapor deposition (PVD) have been widely used to form conductive networks on fibers [28,29,30]. However, due to the intrinsic insulating nature of fibers and their flexible, deformable structures, these surface-based conductive layers often suffer from poor adhesion, mechanical instability, and susceptibility to fracture or delamination under bending, twisting, or stretching. Consequently, long-term durability and performance stability remain challenging, particularly in wearable and flexible applications. Atomic-layered-deposition (ALD), in contrast, provides excellent conformality, enabling uniform deposition of conductive materials even on complex three-dimensional porous fiber architectures [31,32]. While most existing ALD processes for metals have been developed on rigid substrates requiring high temperatures [33,34], flexible textiles are temperature-sensitive and thus may degrade at elevated temperatures. Therefore, developing low-temperature ALD techniques is critical for constructing stable, mechanically robust, and wearable conductive networks, which is the approach adopted in this work. Thus, achieving intrinsic fiber-level conductivity and strong interfacial coupling with MXene is crucial for high-performance wearable electronics [35,36].

In this study, we address these challenges by introducing atomic-layer-grown Pt as a conformal, intrinsically conductive [37,38,39], and MXene-compatible interfacial layer on fabric substrates. An ozone plasma–enhanced atomic layer deposition (PE-ALD) process was used to activate oxygen species on textile surfaces [40,41,42,43], enabling efficient Pt nucleation and high-quality film growth at a low temperature of 100 °C—fully compatible with thermally sensitive fibers (compare with the traditional silicon base) [44,45]. This method yields uniform Pt films with a growth rate of 0.55 Å per cycle, excellent smoothness (Root Mean Square (RMS) = 0.2 nm), high purity, and low resistivity (14 μΩ·cm at 20 nm). Density functional theory (DFT) calculations reveal that ozone activation significantly enhances Pt adsorption energy on fabric-derived carbon sites by modulating differential charge distribution, thereby promoting strong nucleation and bonding. The conformal Pt coating endows the fibers with intrinsic conductivity and greatly enhances the adsorption and interfacial charge transport of MXene gel inks, resulting in a stable conductive network that maintains performance under bending and stretching. Leveraging these advantages, the MXene/Pt@textile demonstrates high sensitivity for detecting human physiological signals—including pulse, vibration, and bending—and functions as an efficient electrode for triboelectric nanogenerators (TENGs) with output voltages up to 18–20 V. Collectively, this work provides a universal low-temperature route for noble metal metallization of fabrics while revealing an effective pathway by which atomic-layer-grown Pt significantly boosts MXene gel ink adsorption and sensing performance, offering a promising platform for next-generation wearable and self-powered electronic systems.

## 2. Results and Discussion

### 2.1. MXene/Pt@Textile: High-Performance Flexible Self-Powered Sensor

**Figure 1:**
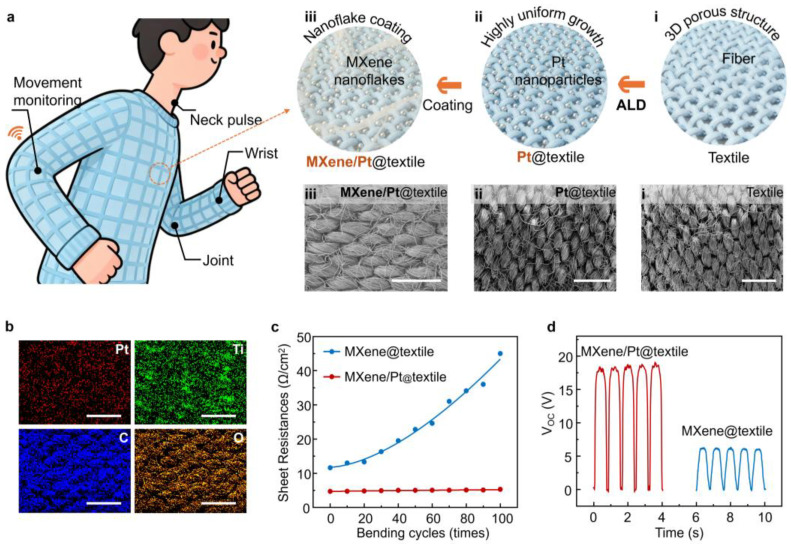
High-performance flexible self-powered sensing textile constructed with Pt and MXene. (**a**) Schematic diagram of Pt deposition and MXene coating on the textile surface and SEM images of (i): pure textile, (ii): Pt@textile and (iii): MXene/Pt@textile. (**b**) (EDS) elemental mapping of the MXene/Pt@textile surface, showing the homogeneous distribution of Pt, Ti, C, and O elements after Pt deposition and MXene coating. (**c**) Sheet resistance comparison of MXene@textile and MXene/Pt@textile after 0–100 twisting cycles. (**d**) Open-circuit voltage (V_OC_) output of MXene@textile and MXene/Pt@textile-based TENG. The scale bars are 1 mm in (**a**,**b**).

Figure 1a illustrates the Pt atomic enhanced conductive fabric strategy developed to address the weak interfacial adhesion of MXene nanoflakes and the poor electrical conductivity of textile substrates. Leveraging the excellent conformality and uniformity of ALD, ultrathin Pt layers were grown directly on textile fibers. The resulting Pt composite textile deposited on textile enables dual functionality: physiological signal detection when attached to the wrist and motion monitoring when integrated into clothing. Schematic and scanning electron microscope (SEM) images on the right side of Figure 1a further compare i: pristine textile, ii: ALD Pt nanoparticles deposited on textile (Pt@textile), and iii: Ti_3_C_2_T_x_ MXene nanoflakes coating on the Pt@textile (MXene/Pt@textile), and the corresponding Energy-dispersive spectroscopy (EDS) mapping are shown in Figure S1, Figure S2, and Figure 1b, respectively. The EDS of the pristine textile (Figure S1) clearly shows only the intrinsic C and O signals originating from the fiber substrate. After Pt deposition, the Pt@textile sample (Figure S2) exhibits a uniform and continuous Pt elemental distribution across the entire fiber surface, demonstrating that the ALD process can effectively construct a dense Pt nanolayer on the textile. Furthermore, the EDS mapping of the MXene/Pt@textile sample in Figure 1b reveals the homogeneous distribution of Pt, Ti, C, and O, confirming that the subsequently sprayed MXene nanoflake gel forms a conformal coating on the Pt@textile surface and results in uniform metallization with improved overall conductivity.

As illustrated in Figure 1c, the electrical conductivity of both MXene-only-coated textile (MXene@textile) and MXene/Pt@textile were tested along with different twisting times. It is evident that the resistance of MXene@textile increases significantly under torsional deformation, rising from 11.6 to 45.0 Ω/cm^2^ after 100 twisting cycles. In contrast, MXene/Pt@textile exhibits much more stable electrical performance and maintains a resistance of approximately only 5.0 Ω/cm^2^ even after 100 twisting cycles. To further correlate the electrical performance with structural stability, additional SEM images were incorporated (Figure S3). MXene/Pt@textile retains its original morphology, with the MXene coating firmly anchored on the fibers. In contrast, MXene@textile shows partial delamination after bending, consistent with its increased resistance. These results confirm that the MXene/Pt composite coating forms a more robust and highly connected conductive network that withstands repeated mechanical deformation. Following the electrical stability analysis, the triboelectric output performance was also evaluated (Figure 1d). The open-circuit voltage increases from 6 V for MXene@textile to 18 V for MXene/Pt@textile, representing more than a threefold enhancement. This demonstrates that in addition to significantly improved electrical conductivity, MXene/Pt@textile also exhibits superior mechanical durability and functional performance in flexible applications.

### 2.2. ALD for Pt Layer

**Figure 2:**
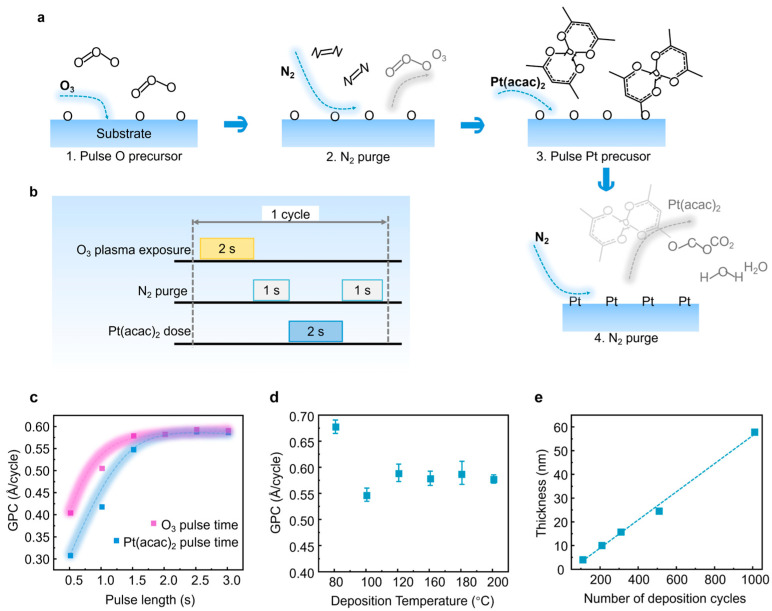
Preparation procedure and growth parameters of ALD Pt growth. (**a**) Schematic representation of the Pt film deposition process via PE-ALD. (**b**) Schematics for the deposition of Pt thin films, showing the pulsed PE-ALD sequences. Pt(acac)_2_ pulses (blue waveform) occur 2 s after the O_3_ plasma pulses (yellow waveform). The precursor’s delivery per subcycle consists of 2 × 1 s pulses. (**c**) Growth rate, measured with X-ray reflectivity (XRR) of Pt film on Si (111) substrates as a function of Pt(acac)_2_ and ozone pulse length pulse length. A total of 1000 cycles were applied at 140 °C. (**d**) Growth rate of Pt film on Si (111) substrates as a function of deposition temperature. Pulse lengths were 2 and 1 s for Pt(acac)_2_ and ozone, respectively. A total of 1000 cycles were applied in each process. (**e**) Thickness of Pt as a function of number of deposition cycles. Pulse lengths for Pt(acac)_2_ and ozone were 2 and 1 s, respectively. The films were deposited at 140 °C.

The ALD sub-cycling process for Pt deposition is depicted in Figure 2a. It consists of sequential alternating pulses of gaseous chemical precursors that react with the substrate. These individual gas–surface reactions are referred to as ‘half-reactions’ and appropriately constitute only part of the material synthesis. During the first half-reaction, the ozone (O_3_) is pulsed into a chamber under vacuum for a designated amount of time to allow the co-reactant to fully react as an oxidizing agent. The plasma-treated ozone mono-layer adheres to the substrate surface via a self-limiting adsorption process, ensuring that the coverage does not exceed a single molecular layer. This O_3_ plasma enhances the adsorption and reaction ability of O on the substrate, solving the difficulty of Pt nucleation and growth at low temperatures. Subsequently, the chamber is purged with an inert carrier gas, nitrogen, to remove any unreacted precursor or reaction by-products, ensuring a clean surface for the subsequent reaction. Then, the counter-reactant precursor, Pt(acac)_2_, is introduced and reacts with the surface-bound oxygen atoms to generate up to one layer of the required material, Pt. Following this reaction, the reactor chamber is again purged with nitrogen to remove by-products such as carbon dioxide and water [35].

As depicted in Figure 2b, we identified the ideal parameters: a 1 s nitrogen purge follows each 2 s pulse of O_3_ and Pt(acac)_2_. This super-cycle was then reiterated to cultivate films with a targeted thickness of approximately 20 nm. The modifiable nature of each process step facilitates an exacting investigation into the parameters that are instrumental in the high-quality deposition of Pt films. Figure 2c illustrates the impact of adjusting Pt(acac)_2_ and ozone pulse lengths on the growth rate of Pt growth. At a fixed O_3_ pulse length of 2 s, the growth rate is approximately 0.57 Å/cycle for a 2 s Pt(acac)_2_ pulse, escalating marginally to nearly 0.59 Å/cycle with a 3 s Pt(acac)_2_ pulse. The growth rate’s temperature dependence, as seen in Figure 2d, shows a decrease from 0.67 Å/cycle to about 0.55 Å/cycle as the deposition temperature rises from 100 °C to 120 °C. When applying Pt precursors in ALD processes, it is essential to strictly control the temperature range. A lower temperature limit, such as 80 °C, is necessary to ensure that the precursors can effectively sublimate and provide a stable vapor flux. Conversely, temperatures that are too high (>200 °C) may lead to non-self-limiting thermal decomposition, thereby disrupting the ideal layer-by-layer growth mechanism of ALD, ultimately resulting in reduced film purity and increased roughness. As shown in Figure 2d, we confirmed that the growth rate of Pt films remains stable (approximately 0.55–0.67 Å/cycle) within the range of 100 °C to 200 °C, thereby clearly defining the ALD temperature window for Pt deposition. This behavior is bounded by the Pt(acac)_2_ sublimation temperature at 110 °C and the favorable metallic form transition at higher temperatures, similar to that in ALD processes using molecular oxygen. As depicted in Figure 2e, exemplified by a Pt film on a Si (111) substrate with increasing cycling times, a linear relationship is affirmed between film thickness and deposition cycles, substantiating a consistent growth rate of approximately 0.59 Å/cycle.

### 2.3. Surface Morphology

**Figure 3:**
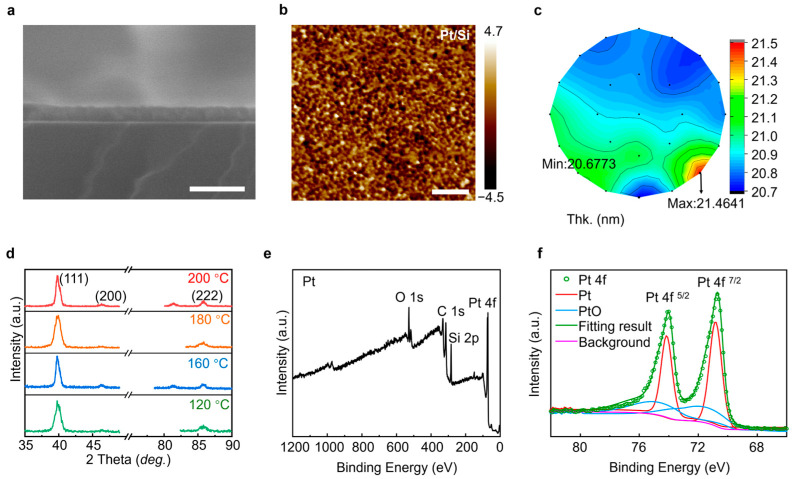
Characterization of Pt film on silicon wafer. (**a**) Cross-section FE-SEM and (**b**) AFM image of Pt film. (**c**) Uniformity testing of Pt films with a thickness of 20 nm on the surface of a six-inch diameter silicon wafer. (**d**) XRD patterns of the films deposited at different temperatures; 1000 cycles were applied in each process. Pulse lengths were 3 and 3 s for Pt(acac)_2_ and ozone, respectively, and the substrate was silicon wafer. (**e**) Representative survey scan. (**f**) Pt 4f core-level XPS spectra for the Pt film, which was grown on silicon substrate. The scale bar is 100 nm in (**a**), 200 nm in (**b**).

In the cross-section SEM image shown in Figure 3a, the thickness of the Pt film is measured at approximately 20 nm, which matches the growth amount per cycle (GPC) of 0.55 Å/cycle. Within the field of view, it can be seen that the film is very flat and the thickness is very uniform, and this structure provides for the excellent adhesion of the coating due to the metallurgical bonding with the substrate. Additionally, Figure S4 presents the cross-sectional SEM Energy Dispersive Spectrometer (EDS) mapping, which provides a detailed visualization of the elemental distribution within the Pt. The field emission scanning electron microscopy (FE-SEM) image in Figure S5a depicts the surface morphology after 1000 sub-cycles of ALD on a silicon substrate. As depicted in Figure S5b, the elemental mapping images illustrate the homogeneous distribution of Pt across the entire silicon wafer, thereby confirming the uniform deposition of Pt facilitated by the interaction between Pt(acac)_2_ and plasma ozone. The RMS roughness was determined to be 0.2 ± 0.01 nm, as illustrated in Figure 3b and Figure S6a,b. The uniformity of the Pt film is exemplified in Figure 3c, where the thickness varies from a maximum of 21.4641 nm at the thickest point to a minimum of 20.6773 nm at the thinnest point. Furthermore, Figure S7 demonstrates that the electrical conductivity of the Pt films is also uniform, ranging from a high of 18 μΩ⋅cm to a low of 14 μΩ⋅cm. Figure S8 displays the peak pattern of the elements as determined by SEM-EDS, where each element appears as a distinct peak.

### 2.4. Crystalline Structure and Chemical State

In addition, Figure 3d shows the XRD patterns of the Pt thin films deposited on Si (111) between 120 and 200 °C. A total of 1000 cycles were applied in each PE-ALD process. The diffraction peaks in their XRD characterizations reveal that the films assisted by ozone are all metallic Pt with face-centered cubic (fcc) structure obtaining orientations of (111) (2θ = 39.2°) and (222) (2θ = 84.3°). In addition, temperatures above 240 °C or below 100 °C do not produce any thin film on any substrates. The metallic Pt films exhibit polycrystalline structure with a strong (111) orientation similar to the films produced by ALD using MeCpPtMe_3_ and molecular oxygen [37].

To elucidate the elemental compositions and structural characteristics of the Pt film, X-ray photoelectron spectroscopy (XPS) was utilized (Figure 3e). In addition, the valence band spectrum is also presented in Figure S9a. All the spectra were calibrated using C–C binding energy at 284.8 eV, which comes from contaminant carbon (Figure S9b). Oxygen adsorption onto the surface of the Pt particles was also confirmed by the presence of the Pt oxide peak at 530.1 eV in the O 1s spectrum in Figure S9c. The Si 2p spectrum distinguished components at 102.3 eV for organic Si and 103.5 eV for SiO_2_ from the base in Figure S9d. The Pt 4f spectra (Figure 3f) showed the major contributing peaks that accompany the minor components at higher binding energy values. The overlapping peaks at 72.5 and 75.83 eV in the spectrum were assigned to Pt oxide, indicating the presence of a minor amount of Pt oxide. And the main peaks at 71.2 and 74.5 eV were attributed to the separated spin-orbit component of the metallic Pt (ΔE = 3.33 eV). The temperature and performances of ALD Pt layer growth are summarized in Table S1.

### 2.5. Theoretical Simulation

**Figure 4:**
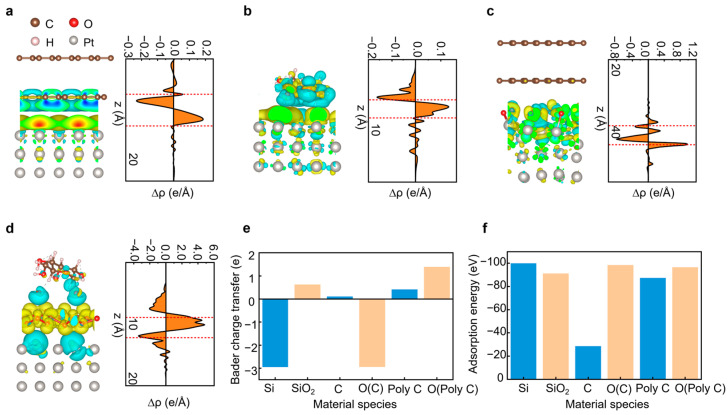
Differential charge and adsorption binding simulations. (**a**) C-Pt structure and its differential charge map (schematic of the molecular structure on the left, schematic of the corresponding adsorption surface differential charge planes on the right, and the dashed line is the molecular adsorption surface reference line). (**b**) C-O-Pt structure and its differential charge map. (**c**) Poly C-Pt structure and its differential charge map. (**d**) Poly C-O-Pt structure and its differential charge map. The yellow and blue areas represent a gain and loss of electrons, respectively. (**e**) Bader charge transfer between seven different material species. (**f**) Adsorption energy of seven different material species.

To further evaluate the how different substrate chemistries influence the nucleation and growth behavior of Pt films during ALD, we conducted first-principles calculations to analyze the molecular configurations and charge transfer characteristics of Pt in contact with various substrate surfaces. Figure 4a–d and Figure S10 depict the molecular structures and corresponding differential charge plots of Pt in contact with various substrate materials. It can be observed that when Pt is in contact with a Si substrate, it exhibits a greater ability to transfer charge, with an electron transfer quantity of 2.9 e^−^. This suggests that Pt atoms may form stronger covalent bonds with Si atoms, resulting in higher adsorption energy of −100.09 eV for the system. In contrast, substrates such as SiO_2_, C, and poly-C (the main components of flexible substrates like fabrics) demonstrate lower electron transfer capabilities and adsorption energies. This is the primary reason why Pt metal struggles to grow into Pt films on these surfaces. As a comparison, O atoms were constructed on the C and poly C substrate surfaces. Figure 4c,d illustrates the binding structures and differential charge plots of O-C and O-poly C with Pt. It is evident that the electron transfer quantity increases significantly from 0.11e (C-Pt) and 0.42 e (Poly-C-Pt) to 2.94e and 1.39e, respectively. Concurrently, the adsorption energy is enhanced from −28.53 eV (C-Pt) and −87.3 eV (Poly-C-Pt) to −98.5 eV and −96.47 eV, respectively. Therefore, pretreating the substrate surface with plasma ozone before Pt film growth can effectively increase the adsorption strength of Pt atoms, thereby yielding high-quality Pt films.

Building upon the above understanding of Pt nucleation and interfacial bonding on modified textile substrates, we further investigated the MXene/Pt@textile heterointerface to clarify its role in enhancing charge transport and triboelectric performance. To elucidate the interfacial mechanism, differential charge density (CDD) analysis of MXene/Pt@textile (Figure S11a) and DFT calculations (Figure S11b) reveals strong electronic coupling between Pt nanoparticles and the MXene nanoflakes surfaces. Both the spatial CDD map and the plane-averaged profile show pronounced charge redistribution—electron accumulation (green and positive peaks) and depletion (yellow and negative peaks), at the MXene/Pt interface. This indicates significant polarization and modulation of Pt electronic states by the MXene nanoflakes.

### 2.6. Wearable Electronic Performance

**Figure 5:**
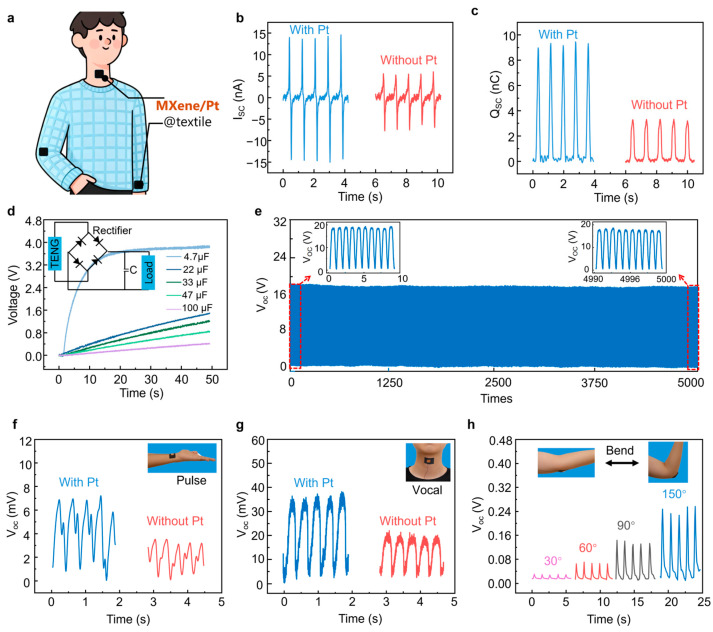
Triboelectricity properties and human biosignal monitoring based on MXene/Pt@textile as electrode. (**a**) Schematic diagram of MXene/Pt@textile worn on the human body. (**b**) Short-circuit current output, and (**c**) short-circuit charge output of MXene@textile and MXene/Pt@textile as the TENG electrode. (**d**) The resulting curves of MXene/Pt@textile-based TENG charging different capacitors. (**e**) Stability of the measured open-circuit voltage for more than 5000 cycles of MXene/Pt@textile electrode. The epidermic sensor assembled from the MXene/Pt @textile for human motion detection. The recorded sensing response of the sensor for (**f**) pulse, (**g**) vocal, and (**h**) arm bending. Inset: photographs of the sensors installed to the radial artery of the wrist, throat, and arm.

To further validate the application of this composite fabric material in wearable electronics, we conducted further investigations on the preparation of MXene/Pt@textile as an electrode for TENGs. At the same time, under the same conditions, MXene@textile was used as a control for comparison. As shown in Figure 5a, the conductive textile can conform intimately to human skin, providing a comfortable and unobtrusive wearing experience. Their energy conversion abilities are compared in Figure 5b,c. It can be clearly seen that MXene/Pt@textile shows a higher electrical output. The short-circuit current (I_sc_) reaches 14 nA, and the short-circuit charge (Q_sc_) reaches 10 nC, both representing nearly 200% improvement compared with MXene@textile without an ALD Pt layer. Figure 5d further demonstrates the charging capability of the MXene/Pt@textile-based TENG for a series of capacitors. The smooth charging profiles and consistent charging times indicate reliable charge generation and transfer during continuous operation. Moreover, compared with MXene@textile (Figure S11), the MXene/Pt@textile-based TENG shows substantially accelerated charging. For example, it charges a 4.7 μF capacitor to 3.8 V within 50 s, whereas MXene@textile only reaches 2.7 V under the same conditions. This pronounced difference directly highlights the enhanced instantaneous power output and sustained energy-harvesting capability of the MXene/Pt@textile-based TENG, underscoring its promise for diverse self-powered applications. Mechanical durability was also assessed by monitoring the output voltage during 5000 repeated contact–separation cycles at 5 Hz and 7 N (Figure 5e). The voltage signal remains nearly unchanged throughout the test, confirming the excellent mechanical and electrical robustness of the device.

Furthermore, when used it as a single-electrode sensor, the MXene/Pt@textile enables highly sensitive and stable monitoring of various human biosignals. As shown in Figure 5f–h, attaching the e-textile to the wrist, throat, and elbow enables real-time detection of heart pulses, speech-induced vibrations, and elbow-bending angles, respectively. With Pt deposition, the sensing sensitivity of the conductive textile is markedly improved, resulting in nearly 200% higher output voltage under identical conditions. In particular, Figure 5h demonstrates that the device can accurately distinguish different bending angles of the elbow, revealing its potential for wearable human-motion monitoring systems.

## 3. Conclusions

In this work, we address two long-standing challenges in wearable electronic textiles: the weak interfacial adhesion between MXene nanoflakes and textile substrates, and the insufficient conductivity of conventional conductive textiles during dynamic deformation. By integrating an ozone-activated, low-temperature ALD strategy, we achieve uniform atomic-layer growth of Pt on porous textiles at only 100 °C. Ozone plasma activation introduces abundant surface oxygen functionalities, significantly improving precursor adsorption and enabling complete surface reactions, thereby yielding high-purity Pt films with excellent continuity on thermally sensitive fibers. The Pt-modified textile forms a robust and conductive scaffold that strengthens MXene adsorption, enhances interfacial charge transport, and markedly reduces resistance fluctuation under mechanical stress. Benefiting from these synergistic effects, the MXene/Pt@textile exhibits dramatically improved triboelectric output, achieving nearly 200% enhancement in short-circuit charge and current compared with unmodified MXene textiles. The MXene/Pt@textile-based TENG also delivers efficient capacitor charging, long-term operational stability over 5000 cycles, and high sensitivity in detecting multiple human physiological signals—including pulse, throat vibration, and joint bending—demonstrating its potential for real-time wearable sensing. Overall, this study provides a universal and substrate-compatible route for noble-metal atomic-layer growth on deformable fabrics, enabling simultaneous improvements in adsorption, conductivity, and sensing performance. The proposed MXene/Pt@textile offers a versatile platform for next-generation wearable electronics, self-powered sensing, and intelligent human–machine interfaces.

## 4. Materials and Methods

### 4.1. Materials and Instruments

The ALD process was conducted using an Elegant II-Y from ANaME, Nanjing, China. High-purity nitrogen (N_2_) served as both the purging and carrier gas, while ozone was generated using a Nanofrontier XLK-G20 ozone generator (Shandong Zhiwei Environmental Protection Technology Co., Ltd., Zhucheng, China). The reaction chamber was maintained at temperatures ranging from 100 °C to 200 °C, with an argon (Ar, 99.9997%) flow rate of 60 sccm through the delivery lines. The Pt precursor, Pt(acac)_2_ (Sigma-Aldrich, St. Louis, MA, USA, 99.999%), served as the source for Pt, and ozone was utilized as the co-reactant in each ALD cycle. The layered 2D Ti_3_C_2_T_x_ MXene gel ink was prepared by selective etching of Al atomic layers from a Ti_3_AlC_2_ MAX phase precursor. The precursor particles were in bulk with an average size of 400 mesh (~40 μm), which was purchased from 11 Technology Co., Ltd., Changchun, Jilin, China. The commercial textiles were purchased from Ruyi Color Fabric Store, Guangzhou, China.

### 4.2. ALD Process

Substrates underwent a cleaning process involving sequential rinses with deionized water, ethanol, acetone, and isopropyl alcohol, followed by drying with a nitrogen blow. The Pt(acac)_2_ precursor was kept at 80 °C, and the chamber walls were heated to 120 °C to prevent condensation. The ALD cycle was meticulously designed with the following steps: a nitrogen (N_2_) pulse to establish the carrier gas, an ozone pulse, a subsequent N_2_ purge, and a Pt(acac)_2_ precursor pulse, concluding with a final N_2_ purge to ensure the thorough removal of by-products and any excess precursor. The durations of these pulses were fine-tuned based on previous research to facilitate uniform film deposition. The films were deposited at temperatures ranging from 100 °C to 200 °C to investigate the impact of low-temperature processing on the thin films’ characteristics.

### 4.3. Preparation of MXene/Pt@ Textile

The classic commercial elastic textile was hand-washed with detergent and then ultrasonically cleaned with anhydrous ethanol and deionized water for 20 min each time. The cleaned textile was then placed into the ALD chamber for Pt deposition. The Pt deposition process as discussed in 4.2. Then, the Pt-coated textile was sprayed with 2D MXene nanosheetflakes ink. The Ti_3_C_2_T_x_ MXene nanoflake ink was prepared by using a selective etching method according our previous study [46,47,48]. Each spraying should ensure uniformity and wetting. After each spraying step, the textile was dried at 60 °C.

### 4.4. Characterization

XPS was carried out with a PHI VersaProbe II Scanning XPS Microprobe, Chanhassen, MN, USA. XPS characterized the compositions and electronic structures of the resulting films. The elemental spectra of O 1s, C 1s, and Pt 4f and the valence spectra were measured. All XPS data were then processed using CasaXPS 2.3.26 software. Crystallinity and phases of Pt films were identified by Grazing Incidence X-ray Diffraction (GIXRD) using a SmartLab with advanced powder X-ray Cu Kα radiation diffractometry with high-resolution parallel-beam optics from Rigaku, Tokyo, Japan. A 1.5 kW copper tube operated at 20 kV and 2 mA, and a 2θ scan was carried out from 5 to 90°. A GeminiSEM 300 Hz high-resolution field emission FE-SEM from Japan was used to characterize both film thickness and surface morphology for before and after annealing. The uniformity testing was performed by ME-Mapping from Wuhan E-Optics Technology Co., Ltd, Wuhan, China. AFM images of Pt films deposited on Si substrates were characterized using a Bruker Dimension Icon scanning probe microscope from Bruker, Milton, CA, USA.

### 4.5. Power Generation Test

The short-circuit current was measured with an SR570 current preamplifier, Stanford Research System, Sunnyvale, CA, USA. The short-circuit transferred charges and the open-circuit voltage were both measured with a Keithley 6514 electromete, Beaverton, OR, USA. The test area was 4 cm^2^, the test frequency was 5 Hz, the test time was 1000 s, and a cyclic compression force of 7 N was used.

## Data Availability

Data will be made available on request.

## Supplementary Materials

The following supporting information can be downloaded at: https://www.mdpi.com/article/10.3390/gels12010019/s1, Figure S1. Energy-dispersive spectroscopy (EDS) elemental mapping of the pristine textile surface showing only the intrinsic C and O elements of the fiber substrate. The scale bar is 1 mm; Figure S2. Energy-dispersive spectroscopy (EDS) elemental mapping of the Pt@textile surface, demonstrating a uniform and continuous distribution of Pt across the textile fibers, together with the inherent C and O signals. The scale bar is 1 mm; Figure S3. SEM images of (a) MXene@textile, (b) MXene/Pt@textile before and after 100 bending cycles at different magnifications; Figure S4. Elemental mappings for Si, O and Pt of cross–section of the platinum film; Figure S5. (a) FESEM and (b) elemental mapping images of the Pt surface; Figure S6. (a) 3D AFM image of platinum film. (b) Height curve shows the cross-sectional plot of Pt nanoparticles along the blue line in (a); Figure S7. Square resistance test of a platinum film with a thickness of 20 nm on the surface of a silicon wafer with a side length of 7 cm; Figure S8. Elemental distribution of energy scattering spectra (EDS) of Pt films on silicon wafers; Figure S9. (a) ultraviolet photo-electron spectroscopy (UPS) test plot of a 20 nm thick Pt film deposited on a silicon substrate. (b) C 1s, (c) O 1s, (d) Si 2p core-level XPS spectra for the Pt film, which was grown on silicon substrate; Figure S10. Si-Pt structure and its differential charge map. Schematic of the molecular structure on the left, schematic of the corresponding adsorption surface differential charge planes on the right, and the dashed line is the molecular adsorption surface reference line; Figure S11. (a) Molecular model of MXene upon adsorption with Pt and its corresponding differential charge transfer diagram. (b) Energy band structure of Pt atoms adsorbed on Ti_3_C_2_T_x_ MXene; Figure S12. The result curves of MXene@textile based TENG charging different capacitors; Table S1. Summarizing temperature and performances of ALD Pt growth.

## Abbreviations

The following abbreviations are used in this manuscript:

Pt	platinum
ALD	atomic layer deposition
DFT	density functional theory
XPS	X-ray photoelectron spectroscopy
TENG	triboelectric nanogenerator
SEM	scanning electron microscopy
GPC	growth amount per cycle
Pt@textile	Pt nanoparticles deposited on textile
MXene/Pt@textile	MXene nanoflakes coated on Pt@textile
MXene@textile	MXene nanoflakes coated on textile
