# Supplementary material for: Atomic-Layer-Grown Pt on Textile Boosts Adsorption and Sensitivity of MXene Gel Inks for Wearable Electronics
Source: Gels. 2025 Dec 24;12(1):19. doi: 10.3390/gels12010019 (PMC12841041; doi:10.3390/gels12010019)
Supplement: Supplementary file 1 [file gels-12-00019-s001.zip › gels-4024643-supplementary.pdf]

Supporting Information

Atomic-Layer-Grown Pt on Textile Boosts Adsorption and Sensitivity of MXene Gel Inks for Wearable Electronics

Jiahui Li [1], Yang Zhang [1], Weidong Song [1], Zhangping Jin [1], Tao Lan [1], Qiuwei Shi [2,*] and Yannan Xie [1,*]

[1] State Key Laboratory of Flexible Electronics and Information Displays & Institute of Advanced Materials (IAM), College of Materials Science and Engineering, Nanjing University of Posts & Telecommunications, Nanjing 210023, China; iamjhli@njupt.edu.cn (J.L.); 18762267734@163.com (Y.Z.); 1223066533@njupt.edu.cn (W.S.); 1223066444@njupt.edu.cn (Z.J.); 2022060718@njupt.edu.cn (T.L.)

[2] College of Chemistry and Materials Science, Nanjing University of Information Science & Technology, Nanjing 210023, China

* Correspondence: sqw@nuist.edu.cn (Q.S.); iamynxie@njupt.edu.cn (Y.X.)

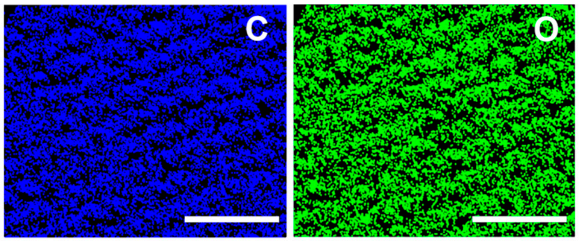

Figure S1 Energy-dispersive spectroscopy (EDS) elemental mapping of the pristine textile surface showing only the intrinsic C and O elements of the fiber substrate. The scale bar is 1 mm.

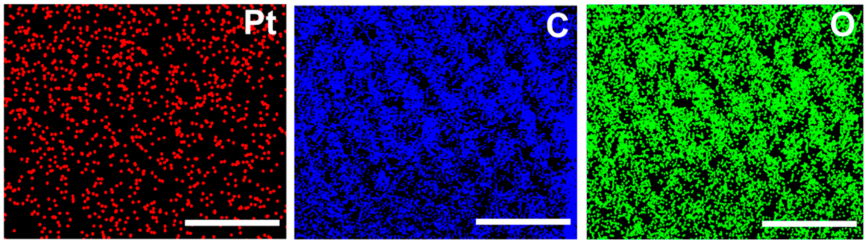

Figure S2 Energy-dispersive spectroscopy (EDS) elemental mapping of the Pt@textile surface, demonstrating a uniform and continuous distribution of Pt across the textile fibers, together with the inherent C and O signals. The scale bar is 1 mm.

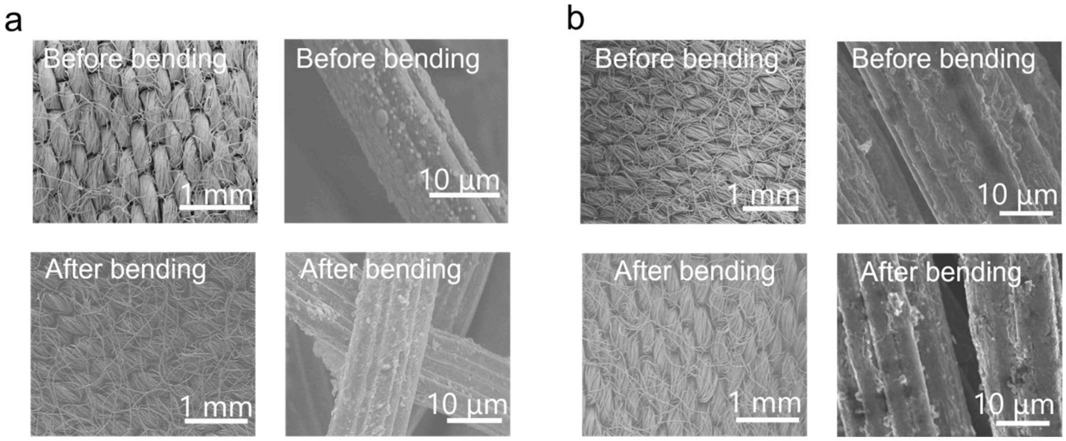

Figure S3. SEM images of (a) MXene@textile, (b) MXene/Pt@textile before and after 100 bending cycles at different magnifications

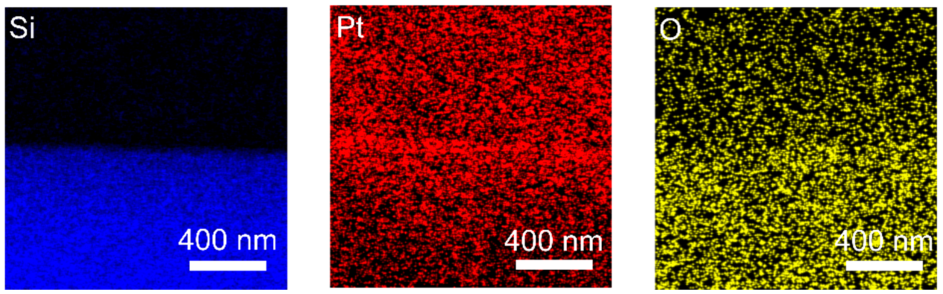

Figure S4 Elemental mappings for Si, O and Pt of cross-section of the platinum film.

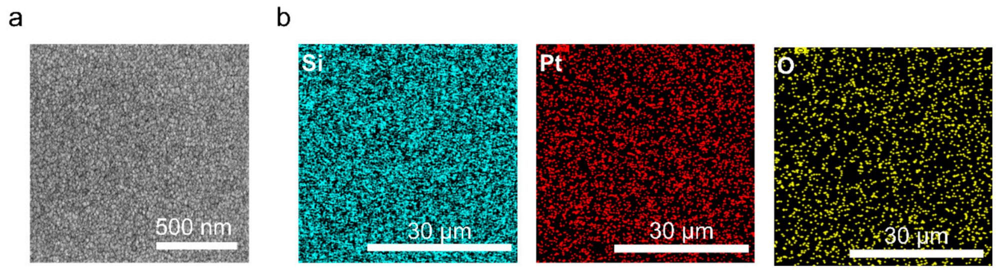

Figure S5. (a) FESEM and (b) elemental mapping images of the Pt surface.

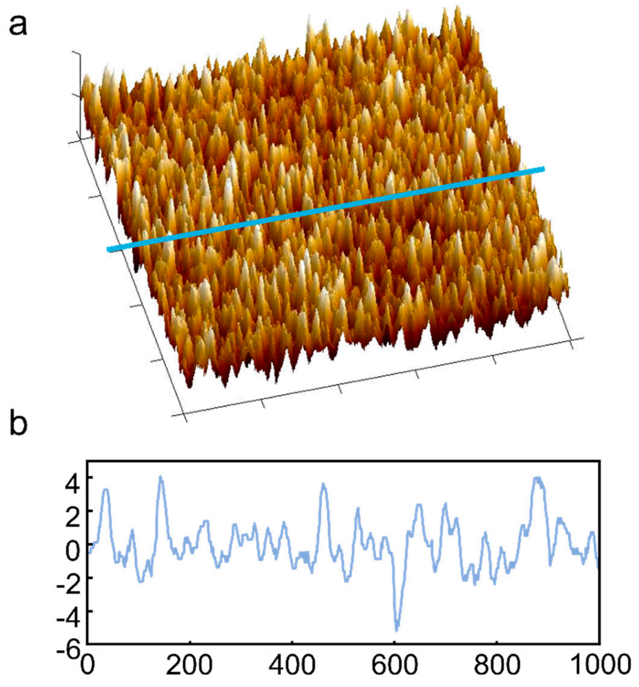

Figure S6. (a) 3D AFM image of platinum film. (b) Height curve shows the cross-sectional plot of Pt nanoparticles along the blue line in (a).

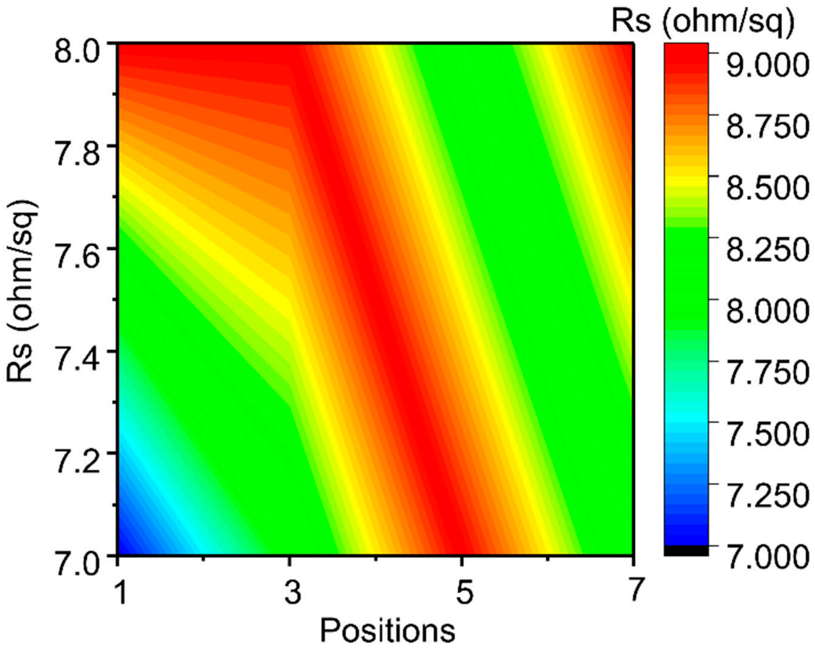

Figure S7. Square resistance test of a platinum film with a thickness of 20 nm on the surface of a silicon wafer with a side length of 7 cm.

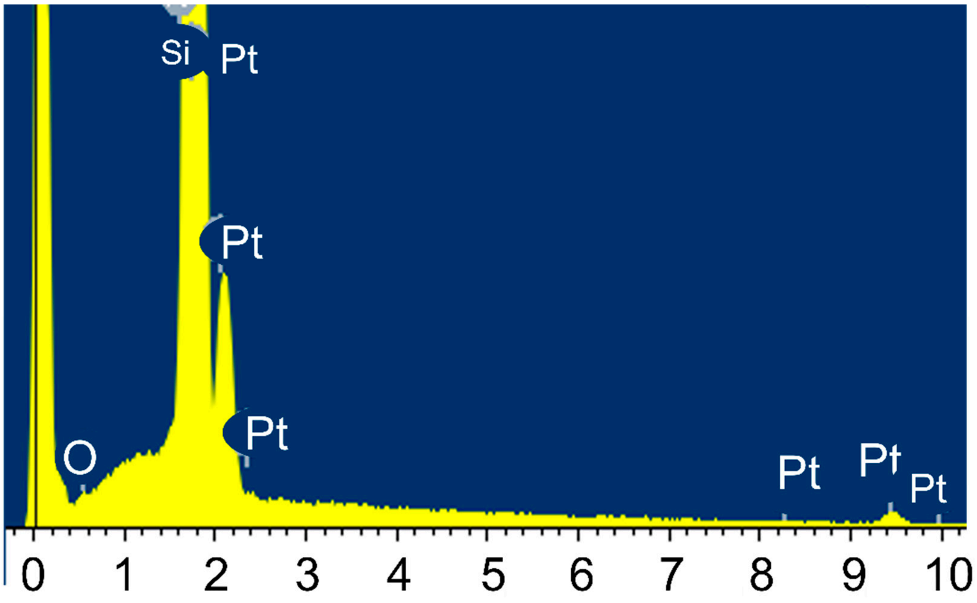

Figure S8. Elemental distribution of energy scattering spectra of Pt films on silicon wafers.

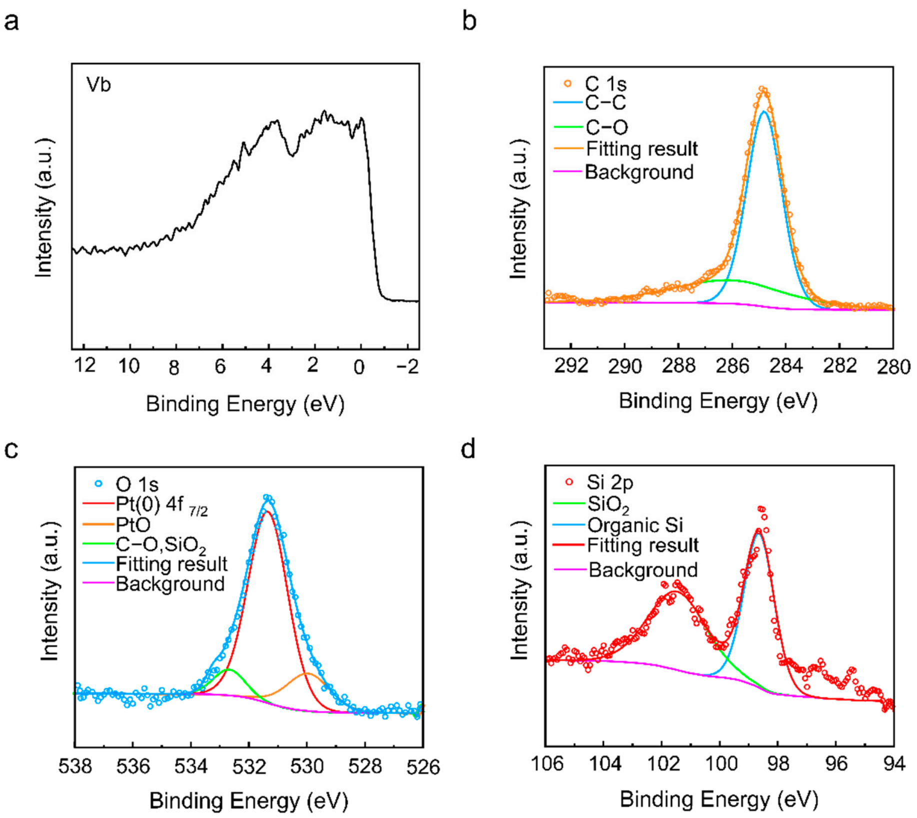

Figure S9. (a) Ultraviolet photo-electron spectroscopy (UPS) test plot of a 20 nm thick Pt film deposited on a silicon substrate. (b) C 1s, (c) O 1s, (d) Si 2p core-level XPS spectra for the Pt film, which was grown on silicon substrate.

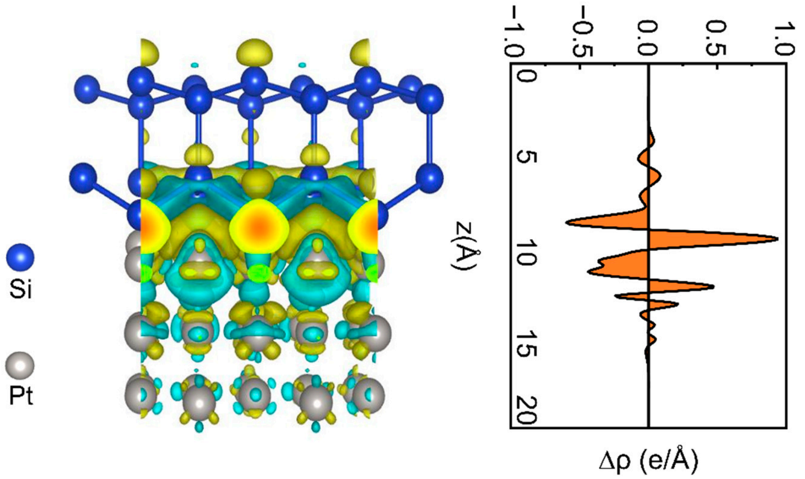

Figure S10. Si-Pt structure and its differential charge map. Schematic of the molecular structure on the left, schematic of the corresponding adsorption surface differential

charge planes on the right, and the dashed line is the molecular adsorption surface reference line.

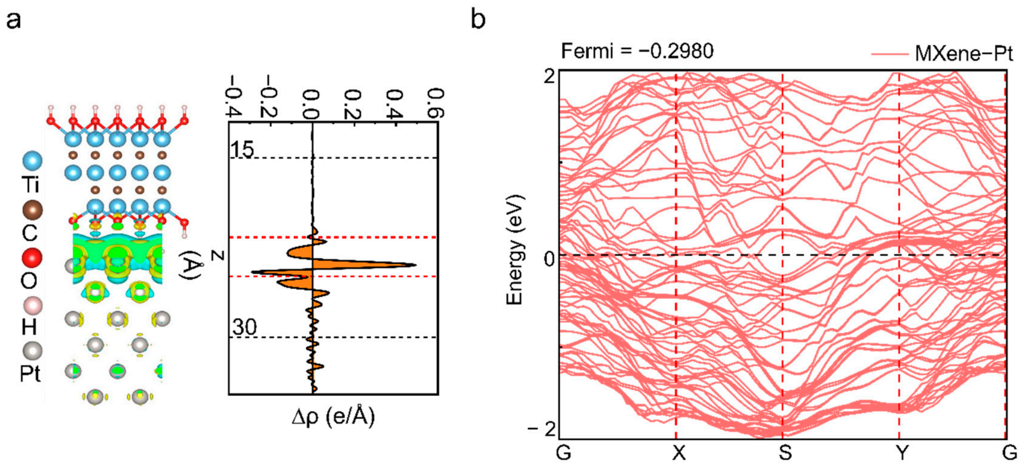

Figure S11. (a) Molecular model of MXene upon adsorption with Pt and its corresponding differential charge transfer diagram. (b) Energy band structure of Pt atoms adsorbed on $Ti_3C_2T_x$ MXene.

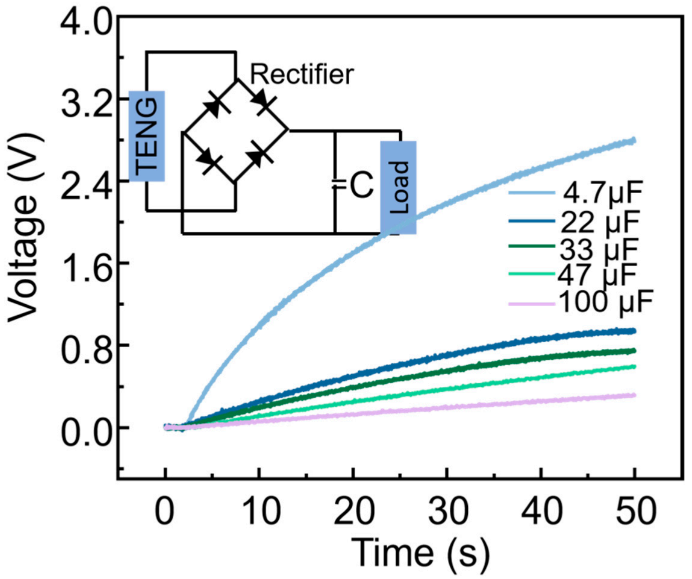

Figure S12. The result curves of MXene@textile based TENG charging different capacitors.

Table S1 Summarizing temperature and performances of ALD Pt growth.

Temperature（°C）	GPC（Å/cycle）	Resistivity (μΩ · cm)	RMS (nm)
100	0.67	14	0.22
120	0.59	15	0.20
150	0.57	16	0.21
200	0.55	18	0.25
